# Methylation analysis in urine fractions for optimal CIN3 and cervical cancer detection

**DOI:** 10.1016/j.pvr.2020.100193

**Published:** 2020-03-13

**Authors:** Rianne van den Helder, Nienke E. van Trommel, Annina P. van Splunter, Birgit I. Lissenberg-Witte, Maaike C.G. Bleeker, Renske D.M. Steenbergen

**Affiliations:** a Amsterdam UMC, Vrije Universiteit Amsterdam, Department of Pathology, Cancer Center Amsterdam, Amsterdam, the Netherlands; b Antoni van Leeuwenhoek/Netherlands Cancer Institute, Department of Gynecologic Oncology, Centre of Gynecologic Oncology Amsterdam, Amsterdam, the Netherlands; c Amsterdam UMC, Vrije Universiteit Amsterdam, Department of Epidemiology and Biostatistics, Amsterdam, the Netherlands

**Keywords:** DNA Methylation, Urine, Comparative analysis, Cervical intraepithelial neoplasia, Cervical cancer

## Abstract

**Introduction:**

Urine sampling is an interesting solution for CIN3 and cervical cancer detection. Urine can be separated in different fractions: full void urine, urine sediment and urine supernatant. We aimed to determine which urine fraction is most competent for CIN3 and cervical cancer detection by methylation analysis.

**Methods:**

Urine samples (27 controls, 30 CIN3 and 17 cervical cancer) were processed into 3 fractions and tested for 5 methylation markers (*ASCL1*, *GHSR*, *LHX8*, *SST*, *ZIC1*). We determined Spearman correlation coefficients between fractions, compared methylation levels and calculated AUCs for CIN3 and cancer detection.

**Results:**

In general strong correlations (r > 0.60) were found between urine fractions. Methylation levels increased significantly with severity of underlying disease in all urine fractions. CIN3 and controls differed significantly for 2 markers in full void urine, 4 markers in urine sediment and 1 marker in urine supernatant, with AUCs of 0.55–0.79. Comparison of cancer to controls was highly significant for all markers in all fractions, yielding AUCs of 0.87–0.99.

**Conclusion:**

Methylation analysis performs excellent in all urine fractions for cervical cancer detection. Our results indicate the potential of CIN3 detection by urinary methylation analysis, and demonstrate that urine sediment performs best to detect CIN3.

## Highlights

• Methylation analysis in urine is attractive for CIN3 and cervical cancer detection.
• Full void, sediment and supernatant were tested for 5 methylation markers.
• All fractions perform excellent for cervical cancer detection.
• Urine sediment performs best to detect CIN3.
• Urinary methylation analysis has potential for CIN3 detection.

## 1 Introduction

Cervical cancer is the fourth most common cancer in women worldwide [1]. Cervical cancer is caused by a persistent infection with high-risk human papillomavirus (hrHPV) and develops through cervical intraepithelial neoplasia (CIN: graded 1 to 3) over a long term period. The incidence of cervical cancer can be reduced by (population-based) screening for high-grade CIN (CIN2 and CIN3) lesions. The participation rate is an important factor in the effectiveness of a screening program. Half of cervical cancer cases are being diagnosed in the unscreened population, indicating that screening attendance remains a focus of concern [2,3]. Providing cervicovaginal self-samples for non-responders can increase the participation rate to screening [4,5]. Therefore, with the introduction of primary hrHPV testing in The Netherlands in 2017, cervicovaginal self-sampling is also being offered to non-responders [6]. Recent data however indicate that still more than one third of the Dutch population eligible for cervical screening remains unscreened [7]. Urine sampling is an attractive alternative to other sampling methods, due to its non-invasive and easy accessible manner of sampling and suggested preference over a physician taken cervical scrape or cervicovaginal self-sampling [[8], [9], [10]]. Therefore, urine can be an ideal solution to reach non-responders in screening programs and may also be of interest for clinical practice.

hrHPV testing on urine is promising and it is expected that urine can also be used for the detection of other biomarkers [[11], [12], [13]]. Moreover, our recent data showed that urine samples are suitable for cervical cancer detection by methylation analysis [14]. In this study we demonstrated that both full void urine and urine sediment perform well for cancer detection. However, these data cannot be directly extrapolated to CIN3 detection, which is more challenging.

Therefore, the aim of this study was to systematically compare the diagnostic potential of methylation analysis in three urine fractions (full void, sediment and supernatant) to distinguish between healthy controls, women with CIN3 lesions and cervical cancer.

## 2 Methods

### 2.1 Study population and urine collection

In this study a total of 74 urine samples were included, collected by healthy female controls (n = 27), women diagnosed with a CIN3 lesion (n = 30) and women with cervical cancer (n = 17). All women were instructed to collect a complete urine void, irrespective of time of collection and personal hygiene.

Urine samples from healthy female controls were collected within the Urine Controls (URIC) Biobank from women with a median age of 43 (range: 30–60), without knowledge on their previous or current HPV status. The samples from women with a CIN3 lesion of the cervix were collected within the SOLUTION 2 study. Median age was 38 (range: 23–60) and urine was provided before they received a large loop excision of the transformation zone (LLETZ). The CIN3 lesion was histological confirmed in resected tissue during LLETZ. Urine samples from women diagnosed with a cervical carcinoma were collected within the SOLUTION 1 study. Median age was 51 (range: 37–81) and urine was provided after confirmation of diagnosis and prior to primary treatment. Fifteen women were diagnosed with a squamous cell carcinoma of the cervix and two women with a adenocarcinoma of the cervix (FIGO Ib1–IIIb).

Ethical approval was provided by the Medical Ethical Committee of the VU University Medical Centre for the use of samples collected within the URIC biobank (no 2018.657), the SOLUTION 2 study (no 2017.112) and for the SOLUTION 1 study (no 2016.213) (Trial registration ID: NL56664.029.16). All women provided written informed consent.

### 2.2 Urine processing

Urine samples were collected in three 30 mL collection tubes, each prefilled with 2 mL 0.6 M Ethylenediaminetetraacetic acid (EDTA) resulting in a final concentration of 40 mM. EDTA maintains DNA quality during transport [15]. Collection tubes were sent by regular mail to the pathology department of Amsterdam UMC, VU University Medical Centre, and processed within 24–72 h after collection. Urine sediment and supernatant were obtained by centrifugation of 15 mL of urine at 3000×*g* for 15 min. All three fractions, full void, sediment and supernatant were stored at −20 °C.

### 2.3 DNA isolation and DNA modification

DNA was isolated from urine sediment (15 mL original volume) using the DNA mini and blood mini kit (Qiagen, Hilden, Germany). DNA was isolated from urine supernatant (15 mL original volume) and full void urine (30 mL original volume) using the Quick DNA urine kit (Zymo Research, Irvine, CA, US). A NanoDrop 1000 (ThermoFisher Scientific, Waltham, MA, US) was used for DNA concentration measurements and the 260/280 ratio was determined to assess DNA purity [16]. Isolated DNA was bisulphite converted using the EZ DNA Methylation Kit (Zymo Research, Irvine, CA, US). All procedures were performed according to the recommendations of the manufacturer.

### 2.4 Host cell gene DNA methylation analysis by quantitative methylation specific PCR (qMSP)

Methylation analysis of *ASCL1, GHSR, LHX8, SST* and *ZIC1* was performed in two different multiplex assays (multiplex 1 *GHSR, SST, ZIC1*; multiplex 2 *ASCL1*, *LHX8)* as described before [17,18]. qMSPs were performed using 50 ng of bisulphite-converted DNA. For quantification and quality control *ACTB* was used as a reference gene. Samples with a quantification cycle (Cq) value of *ACTB* > 32 were excluded from methylation analysis to assure good sample quality. The methylation levels of all markers were normalized to reference gene *ACTB* using the comparative Cq method (2^−ΔCq^ x 100) to obtain Cq ratios [19].

### 2.5 Data analysis

For the comparison of DNA concentrations between urine fractions, outliers, i.e. samples with unreliably high DNA concentrations and 260/280 ratio's below 1, were excluded. Concentrations were compared using the Kruskal-Wallis test, followed by pairwise Mann-Whitney U test with Bonferroni correction. For the analysis of methylation levels log2-transformed Cq ratios were used. Correlation between Cq ratios of each methylation marker in unfractioned urine, urine sediment and urine supernatant of women with CIN3 combined with women with cervical cancer (CIN3+) was assessed with Spearman's rank correlation. Differences in methylation levels between control, CIN3 and cervical cancer were visualized using boxplots and tested for statistical significance using the Kruskal-Wallis test, followed by pairwise Mann-Whitney U test with Bonferroni correction (significance: p < 0.025). To determine the potential of *ASCL1*, *GHSR, LHX8*, *SST* and *ZIC1* methylation to discriminate between controls and women with CIN3 or cervical cancer, receiver operating characteristic (ROC) curves were made of all methylation markers in all urine fractions and results were quantified by area under the curve (AUC). All statistical analyses and production of graphs were performed in IBM SPSS 24 and GraphPad Prism 8.

## 3 Results

### 3.1 DNA quantity and quality of urine fractions

To evaluate the utility of full void urine, urine sediment and urine supernatant for methylation analysis we first compared the DNA yield of all urine fractions obtained from urine samples of 27 healthy female controls, 30 women with CIN3 and 17 women with cervical cancer. As shown in Table 1a a wide range in DNA yield per mL urine is found in each of the fractions. Although not significant, in women with CIN3 and cervical cancer the lowest median DNA yield was found in urine sediment, whereas urine supernatant was lowest in healthy controls.

**Table 1a:** DNA quantity characteristics of different urine fractions. Median DNA yield per mL urine including corresponding range. Samples with a 260/280 ratio < 1 were excluded from analysis.

		Full void urine	Urine sediment	Urine supernatant
		ng DNA per mL urine		
Control	median	43.8	29.5	16.9
	min–max	12.0–261.7	6.0–239.0	5.1–306.6
CIN3	median	112.0	76.0	117.0
	min–max	7.0–1134.9	13.0–714.7	17.8–967.0
Cervical cancer	median	106.9	68.6	93.8
	min–max	18.7–332.0	14.3–334.5	13.7–758.2

To further assess the DNA quality for methylation analysis Cq values of the reference gene *ACTB*, as obtained from two independent multiplex qMSP assays, were compared (Table 1b). Cq values of *ACTB* were significantly lower in urine sediment samples compared to full void samples (Multiplex 1 p = 0.007 and Multiplex 2 p = 0.016), as well for supernatant samples (Multiplex 1 p < 0.001 and Multiplex 2 p < 0.001). In line with the lower Cq values in urine sediment, indicating a better quality, only 1 sample tested invalid in urine sediment, compared to 3 and 4 in full void samples and 3 and 5 in supernatant samples.

**Table 1b:** DNA quality characteristics of different urine fractions. Median Cq value of *ACTB* and the percentage of invalid samples for methylation analysis based on a Cq value of *ACTB* > 32.

	Full void urine		Urine sediment		Urine supernatant	
	median Cq	invalid (%)	median Cq	invalid (%)	median Cq	invalid (%)
Multiplex 1 (*GHSR*-*SST*-*ZIC1*)	24.81	3 (4.1%)	24.30	1 (1.4%)	26.63	3 (4.1%)
Multiplex 2 (*ASCL1*-*LHX8*)	25.75	4 (5.4%)	25.35	1 (1.4%)	27.05	5 (6.8%)

### 3.2 Methylation levels in urine fractions

To compare the methylation levels obtained in the different urine fractions, the correlation between paired fractions was determined. For all but one markers a strong to very strong correlation (*r* > 0.60) was found between different urine fractions of women with CIN3+ (Table 2). Only for *GHSR* a moderate correlation was found between urine sediment and supernatant (*r* = 0.59). All correlations were strongly significant (p < 0.001).

**Table 2:** Spearman correlation coefficients (*r*) of all methylation markers between full void urine, urine sediment and urine supernatant of CIN3 and cervical cancer (CIN3+) patients. Samples with a Cq value of *ACTB* > 32 were excluded from analysis. The Spearman correlation coefficient was calculated based on log2-transformed Cq ratio's. All correlations were strongly significant (p < 0.001).

	Full void urine versus Urine sediment	Full void urine versus Urine supernatant	Urine sediment versus Urine supernatant
	CIN3+		
*ASCL1*	0.77	0.82	0.73
*GHSR*	0.77	0.69	0.59
*LHX8*	0.72	0.81	0.76
*SST*	0.64	0.70	0.75
*ZIC1*	0.72	0.80	0.72

r = 0.40–0.59 moderate correlation, r = 0.60–0.79 strong correlation, r = 0.80–1.00 very strong correlation.

### 3.3 Performance of methylation markers urine fractions for CIN3 and cervical cancer detection

**Fig. 1:**
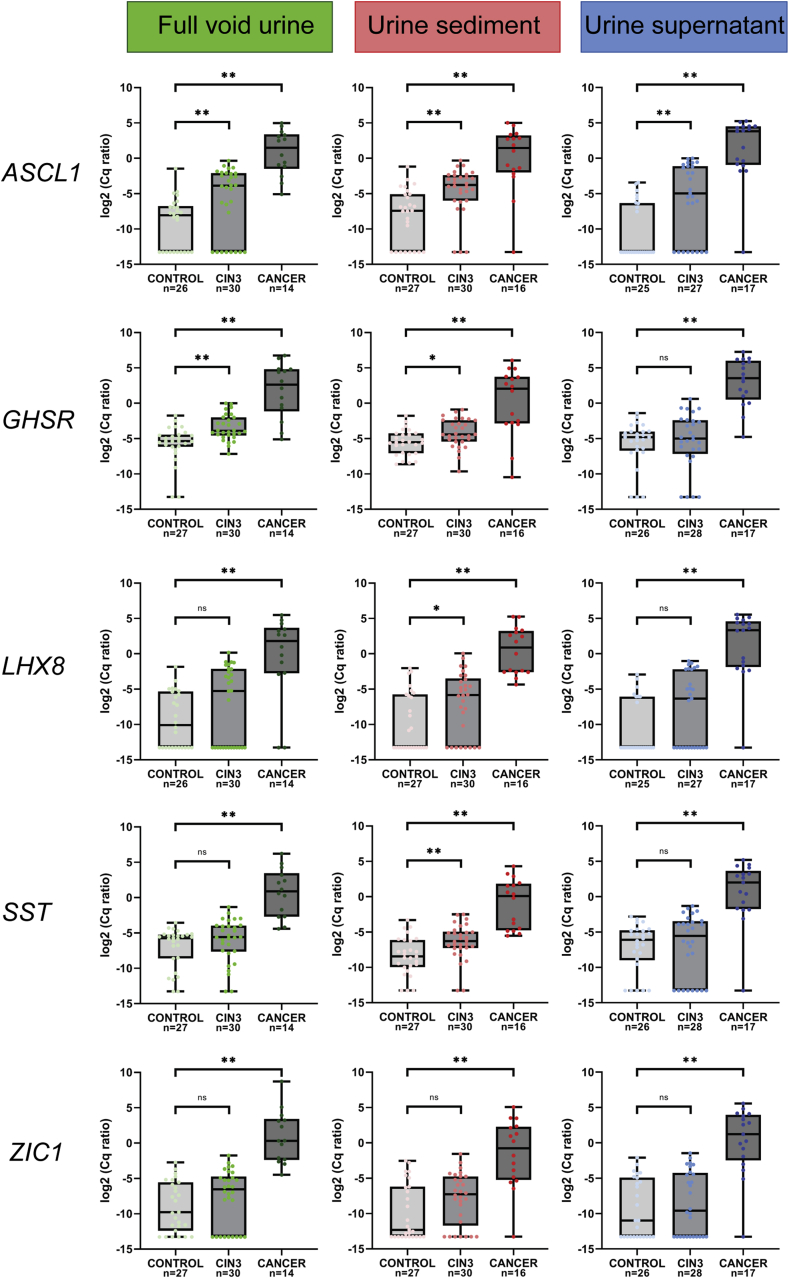
Methylation levels of *ASCL1, GHSR, LHX8, SST* and *ZIC1* in full void urine (green), urine sediment (red) and urine supernatant (blue) from 27 healthy female controls, 30 women with CIN3 and 17 women with cervical cancer. Methylation levels are shown by the log2-transformed Cq ratios. Boxplots show medians with lower and upper quartile and range whiskers. **p < 0.005 * p < 0.025 ns p > 0.025. (For interpretation of the references to colour in this figure legend, the reader is referred to the Web version of this article.)

Methylation levels of all markers increased with increasing severity of disease in all urine fractions (p < 0.001). All methylation markers revealed a significant increase in methylation levels in women with cervical cancer compared to controls for all fractions (Fig. 1). Significantly increased methylation levels in women diagnosed with CIN3 compared to controls were found for two markers (*ASCL1* and *GHSR*) in full void urine, for four markers (*ASCL1*, *GHSR, LHX8* and *SST*) in urine sediment and for one marker (*ASCL1*) in urine supernatant.

To assess which urine fraction has the highest discriminatory potential to distinguish between controls and CIN3 and cervical cancer ROC curves were made and AUCs were calculated (Table 3). For CIN3 detection highest AUC was obtained for *GHSR* (AUC 0.78) in full void urine and for *ASCL1, LHX8*, *SST* and *ZIC1* (AUC 0.62–0.79) in urine sediment. For cervical cancer detection high AUCs were obtained for all fractions (AUC 0.87–0.99).

**Table 3:** The area under the curve (AUC) and 95% confidence interval (95%-CI) of all methylation markers in full void urine, urine sediment and urine supernatant for the detection of CIN3 and cervical cancer. Samples with a Cq of *ACTB* > 32 were excluded from analysis. AUCs in bold represent the fraction in which the specific marker performed best.

	Full void urine	Urine sediment	Urine supernatant
	Control versus CIN3		
*ASCL1*	0.73 (0.59–0.87)	**0.79** (0.67–0.91)	0.75 (0.62–0.89)
*GHSR*	**0.78** (0.67–0.90)	0.70 (0.56–0.84)	0.55 (0.39–0.70)
*LHX8*	0.62 (0.47–0.77)	**0.68** (0.54–0.82)	0.66 (0.52–0.81)
*SST*	0.61 (0.46–0.76)	**0.73** (0.59–0.86)	0.57 (0.41–0.72)
*ZIC1*	0.58 (0.43–0.73)	**0.62** (0.47–0.77)	0.55 (0.39–0.70)
	Control versus Cervical cancer		

*ASCL1*	**0.99** (0.96–1.00)	0.92 (0.80–1.00)	0.96 (0.88–1.00)
*GHSR*	0.95 (0.88–1.00)	0.87 (0.72–1.00)	**0.97** (0.92–1.00)
*LHX8*	0.88 (0.73–1.00)	**0.97** (0.92–1.00)	0.96 (0.88–1.00)
*SST*	**0.99** (0.97–1.00)	0.96 (0.91–1.00)	0.95 (0.84–1.00)
*ZIC1*	**0.99** (0.97–1.00)	0.88 (0.77–1.00)	0.93 (0.84–1.00)

## 4 Discussion

This study is the first to systematically compare host cell gene DNA methylation analysis in different urine fractions for CIN3 and cervical cancer detection. This comparison revealed a good performance in all fractions, including urine supernatant. Based on the sample reliability during testing, best performance in detecting CIN3 and its practical benefits, urine sediment is preferred for CIN3 and cervical cancer detection by urinary methylation analysis.

The existence of a large variation in urine samples both between individuals and between fractions is demonstrated by the large range in DNA yield per mL urine as found in this study. Nevertheless, valid methylation test results were obtained for nearly all individuals and urine fractions. Although urine sediment provided the lowest DNA yield, it appeared to contain the highest amount of amplifiable human DNA based on lowest Cq values of *ACTB.* Accordingly, by using urine sediment less invalid results were obtained. The higher DNA yields combined with the higher Cq values of *ACTB* in full void and supernatant may in part be explained by the presence of non-human DNA (e.g. DNA from the vaginal microbiome).

Snoek et al. compared full void urine versus urine sediment in women with cervical cancer and controls. In both fractions a strong agreement in hrHPV detection and a strong correlation for methylation analysis using six methylation markers was found in women with cervical cancer [14]. This is in line with present observations in full void urine and urine sediment of women with CIN3 and cervical cancer for all methylation markers. Interestingly and unexpected, mostly strong correlations were also found between urine supernatant and both full void urine and urine sediment in women with CIN3+.

These strong correlations are expected to translate to a similar potential for CIN3 and cervical cancer detection in the different urine fractions. Indeed all urine fractions showed excellent performance for cervical cancer detection (AUC 0.87–0.99). The good performance of urine supernatant for cancer detection (AUC 0.93–0.97) may be explained by the presence of cell free DNA [[20], [21], [22]]. More varying results were obtained for CIN3 detection in the different fractions and urine sediment performed best to detect CIN3 (AUC 0.62–0.79). Although, due to the small sample size used for this comparative analysis no conclusions regarding clinical performance can be drawn, we can conclude that urine sediment is the preferred fraction for further studies. Using this knowledge, we are currently extending our sample size. In addition, we will compare urine sediment samples with paired cervicovaginal self-samples and cervical scrapes for both hrHPV detection and methylation analysis. Despite the fact that the use of urine sediment requires an extra centrifugation step, isolation of DNA from urine sediments is less expensive, less labor-intensive and less time-consuming. Furthermore, given the low volume, urine sediments are easier to store. The potential of CIN3 detection by urinary methylation analysis is also supported by two previous studies, which used urine sediment [23] and urine circulating cell-free DNA [24], respectively.

Above studies and also present study used random void urine samples. However, it has been suggested that the use of the first part of the initial urine flow, so-called first-void urine, which also contains exfoliated cells and cell free DNA (e.g. HPV) from the genital tract may be preferred [13,25,26]. This was supported by higher concentrations of hrHPV and human DNA found in first-void urine compared to midstream urine [13,25,27]. Accordingly, first-void urine is expected to be suitable for methylation analysis as well.

## 5 Conclusion

Our comparative analysis shows an excellent performance of all urine fractions for cervical cancer detection using methylation analysis. Furthermore, this study supports the potential of methylation markers to detect CIN3. Urine sediment provides the highest human DNA quality for methylation analysis and the best accuracy to detect CIN3.

## Funding

This work was supported by Stichting Hanarth Fonds, who provided financial support for the conduct of the research and was not involved in conducting the research and preparation of the manuscript.

## CRediT authorship contribution statement

**Rianne van den Helder:** Resources, Investigation, Formal analysis, Visualization, Writing - original draft. **Nienke E. van Trommel:** Funding acquisition, Conceptualization, Resources, Writing - review & editing. **Annina P. van Splunter:** Investigation. **Birgit I. Lissenberg-Witte:** Formal analysis. **Maaike C.G. Bleeker:** Funding acquisition, Conceptualization, Resources, Writing - review & editing. **Renske D.M. Steenbergen:** Funding acquisition, Conceptualization, Methodology, Writing - review & editing, Supervision.

## Declaration of competing interests

Rianne van den Helder, Nienke E. van Trommel, Annina P. van Splunter, Birgit I. Lissenberg-Witte and Maaike C.G. Bleeker have no interests to declare.

Renske D.M. Steenbergen has a minority share in Self-screen B·V., a spin-off company of Amsterdam UMC, location VUmc. Self-screen B.V. holds patents related to the work (i.e., high-risk HPV test and methylation markers for cervical screening) and has developed and manufactured the methylation assay, which is licensed to Qiagen (QIAsure® Methylation Test).
